# Clinical and Laboratory Features of Patients with Multisystem Inflammatory Syndrome in Children (MIS-C): An Experience From Queen Rania Children’s Hospital, Jordan

**DOI:** 10.7759/cureus.37282

**Published:** 2023-04-08

**Authors:** Motasem Alsuwaiti, Raed Alzyoud, Hiba Maaitah, Bushra Aladaileh, Hamzeh Alnsoor, Mohammed Nobani

**Affiliations:** 1 Immunology, Allergy, and Rheumatology, Queen Rania Children's Hospital, Amman, JOR; 2 Immunology, Allergy and Rheumatology, Queen Rania Children's Hospital, Amman, JOR; 3 Immunology, Allergy, and Rheumatology, Queen Rania Children’s Hospital, Amman, JOR

**Keywords:** multi-system inflammatory syndrome in children (mis-c), mis-c in children, jordan, hyperinflammation, multisystem inflammatory syndrome in children, covid-19, mis-c

## Abstract

Background

Multisystem inflammatory syndrome in children (MIS-C) is a new clinical observation that emerged during the coronavirus pandemic of 2019 (COVID-19) and has similar manifestations to Kawasaki disease and toxic shock syndrome. In this study, we aim to describe the characteristics of MIS-C patients in a single center in Jordan.

Methods

A retrospective analysis of electronic medical records of pediatric patients diagnosed with MIS-C at the pediatric rheumatology division of Queen Rania Children’s Hospital, Amman, Jordan, between January 2021 and December 2022. Data collected included age, gender, clinical and laboratory data on presentation, and treatment options, which were compared in two different age groups.

Results

A total of 80 patients were included in this cohort (53 males and 27 females). The mean age at presentation was 84.4 months (ranging between nine months and 16 years). The most common presenting symptoms included fever (100%), abdominal pain (76.2%), skin rash (75%), conjunctivitis (72.5%), and mucosal changes (62.5%). Lymphopenia was present in 66.2% of patients. The majority of patients (98.7%) showed elevated C-reactive protein (CRP); 72 patients showed elevated erythrocyte sedimentation rate (ESR) (92.5%); ferritin was elevated in 70% of patients; the median fibrinogen level was 390 (interquartile range (IQR) 0.6-20) mg/dL; and the D-dimer level was 3.9 (IQR 0.6-20) mg/dL. Pericardial effusion was present in 23.8% of patients, and five patients (6.3%) had coronary artery dilatation.

Conclusion

To the best of our knowledge, this study is the first large case series of MIS-C in Jordan, with a wide spectrum of clinical presentation and evidence of hyperinflammation.

## Introduction

A novel coronavirus called severe acute respiratory syndrome coronavirus (SARS-CoV-2), causing a disease that the World Health Organization (WHO) named "coronavirus disease" (COVID-19), emerged in December 2019 in China [1]. Children were initially thought to be asymptomatic or mildly symptomatic, but different clinical and atypical presentations were reported during the pandemic [2]. Children with features similar to those in Kawasaki disease, Kawasaki disease shock syndrome, and toxic shock syndrome with hyperinflammation were reported by Riphagen et al. [3]. After that, patients with similar symptoms were reported all over the world, and the WHO termed the syndrome a multisystem inflammatory syndrome in children (MIS-C) associated with COVID-19 [4]. The United States Centers for Disease Control and Prevention (CDC) and WHO issued a defining criterion for MIS-C [4,5]. In the absence of previously published reports from Jordan, we report in this study the clinical manifestations and treatment options of children with MIS-C associated with COVID-19 at the Queen Rania Children’s Hospital, Amman, Jordan. We compare the frequency of clinical and laboratory findings in two groups (younger and older than five years of age), which is the age of kindergarten attendance, to find the most common clinical and laboratory manifestations in each age group.

## Materials and methods

A retrospective analysis of medical records of patients younger than 16 years of age diagnosed with MIS-C in the pediatric rheumatology division at Queen Rania Children’s Hospital, Amman, from December 2020 to December 2022 was included in this study. The collected data consisted of demographic characteristics, clinical and laboratory data, and treatment options. The CDC MIS-C definition criteria were used to confirm the diagnosis of MIS-C (Table 1).

**Table 1:** CDC definition criterion of MIS-C i: Fever >38.0°C for ≥24 hours, or report of subjective fever lasting ≥24 hours ii: Including, but not limited to, one or more of the following: an elevated C-reactive protein (CRP), erythrocyte sedimentation rate (ESR), fibrinogen, procalcitonin, d-dimer, ferritin, lactic acid dehydrogenase (LDH), or interleukin 6 (IL-6), elevated neutrophils, reduced lymphocytes and low albumin MIS-C: multisystem inflammatory syndrome in children; SARS-CoV-2: severe acute respiratory syndrome coronavirus 2; RT-PCR: reverse transcription polymerase chain reaction

Definition criterion of MIS-C
Case definition for multisystem inflammatory syndrome in children (MIS-C) by CDC
An individual aged <21 years presenting with fever^i^, laboratory evidence of inflammation^ii^, and evidence of clinically severe illness requiring hospitalization with multisystem (>2) organ involvement (cardiac, renal, respiratory, hematologic, gastrointestinal, dermatologic, or neurological); AND
No alternative plausible diagnoses; AND
Positive for current or recent SARS-CoV-2 infection by RT-PCR, serology, or antigen test; or COVID-19 exposure within the four weeks before the onset of symptoms
Additional comments
Some individuals may fulfil full or partial criteria for Kawasaki disease but should be reported if they meet the case definition for MIS-C
Consider MIS-C in any pediatric death with evidence of SARS-CoV-2 infection

Laboratory evidence of SARS-CoV-2 infection was obtained using either a real-time reverse transcription polymerase reaction (RT-PCR) nasopharyngeal swab or a serology antibody test.

Disease severity was classified as mild (patient with fever and more than two organs involved, stable vital signs, and no signs of cardiac dysfunction; or patient with fever and more than two organs involved, hemodynamically stable with mild cardiac dysfunction); moderate (patient with fever and more than two organs involved, hemodynamically unstable with mild cardiac dysfunction); and severe (patient with fever and two or more organs involved, hemodynamically unstable, or with significant cardiac dysfunction). Cardiac function was assessed by an echocardiogram by a pediatric cardiologist, and the severity was classified based on the shortening fraction (FS), pericardial effusion, and arrhythmia.

Statistical analysis was performed using the IBM Statistical Package for Social Sciences (SPSS) version 26.0 (IBM Corp., Armonk, NY, USA) software package. Percentages were used for qualitative variables, and the mean (SD) was used for quantitative variables. Independent sample t-tests (for two groups) were used to compare numerical data and determine significance. The differences in laboratory parameters between the age groups were analyzed, and a p-value ≤ 0.05 was considered statistically significant in all statistical analyses. A chi-square test was used to compare the categorical data.

Inclusion and Exclusion Criteria

All patients younger than 16 years of age who met the CDC definition criteria for MIS-C were included, and patients with other identified diagnoses or missed follow-ups were excluded.

## Results

A total of 80 patients who met the CDC definition criteria for MIS-C were included in this study. There were 53 males (66.2%) and 27 females (33.8%). The mean age was 84.4 months, and 25 patients (31.2%) were younger than five years.

Twenty-one patients (26.2%) tested positive for COVID-19 via RT-PCR, and 17 (21.1%) had positive serology evidence of COVID-19 infection. Sixty-four patients (80%) reported contact with confirmed COVID-19 patients up to four weeks before presentation. Patient characteristics are demonstrated in Table 2.

**Table 2:** Demographics of 80 patients COVID-19: coronavirus disease 2019; PCR: polymerase chain reaction

Demographics of 80 patients	
Male (%)	53 (66.2%)
Age in months (mean ± SD)	84.4 ± 42.2
COVID-19 PCR-positive	21 (26.2%)
COVID-19 serology-positive	17 (21.1%)
Positive history of contact	64 (80%)

The most common clinical manifestation was fever, which was reported in all 80 patients with a six-day median duration of fever, followed by abdominal pain in 61 patients (76.2%), skin rash in 60 patients (75%), conjunctivitis in 58 patients (72.5%), oral mucosal changes in 50 patients (62.5%), and headache in 31 patients (38.7%).

The patients’ clinical characteristics were stratified by age group and analyzed using a chi-square test, which revealed that abdominal pain was statistically significant in patients over five years of age compared to those younger than five years of age (83.6% versus 60.0%, with a p-value of 0.024). Similarly, headache was statistically significant in patients older than five years compared to those under five years of age (47.3% versus 20.0%, with a p-value of 0.017). However, lymphadenopathy (cervical and axillary) was clinically significant in children under five years of age compared to those older than five years (24.0% versus 7.3%, with a p-value of 0.045) (Table 3).

**Table 3:** Clinical manifestations of MIS-C patients in different age groups FS: fractional shortening

Clinical manifestations of MIS-C patients in different age groups				
Characteristics	Overall 80 (100%)	<5 years 25 (31.25%)	>5 years 55 (68.75%)	p-value
Male (%)	53 (66.2)	16 (64.0)	37 (67.3)	0.483
Female (%)	27 (33.8)	9 (36.0)	18 (32.7)	0.483
Fever (%)	80 (100)	25 (100)	55 (100)	NA
Abdominal pain (%)	61 (76.2)	15 (60.0)	46 (83.6)	0.024
Vomiting (%)	49 (61.2)	13 (52.0)	36 (65.5)	0.184
Diarrhea (%)	34 (42.5)	11 (44.0)	23 (41.8)	0.522
Conjunctivitis (%)	58 (62.5)	18 (72.0)	40 (72.7)	0.574
Mucosal changes (%)	50 (62.5)	18 (72.0)	32 (58.2)	0.176)
Skin rash (%)	60 (75)	20 (80.0)	40 (72.7)	0.344
Peripheral edema (%)	26 (32.5)	8 (32.0)	18 (32.7)	0.581
Headache (%)	31 (38.7)	5(20.0)	26(47.3)	0.017
Drowsiness (%)	5 (20)	5(20.0)	16(29.1)	0.284
Lymphadenopathy (%)	10 (12.5)	6 (24.0)	4 (7.3)	0.045
Coronary artery dilation (%)	5 (6.3)	3 (12.0)	2 (3.6)	0.173
Aneurysm (%)	1 (1.3)	1 (4.0)	00	0.312
Pericardial effusion (%)	19 (23.8)	5 (20.0)	14 (25.5)	0.409
Depressed FS (%)	11 (13.8)	5 (20.0)	6 (10.9)	0.224

Twenty-one patients (26.2%) met the criteria for Kawasaki disease, whereas 31 patients (38.7%) met the criteria for incomplete Kawasaki disease. The clinical manifestations of our cohort are demonstrated in Table 3.

Cardiac involvement was reported in 24 patients (30%), with the most common finding being pericardial effusion in 19 patients (23.8%), which occurred more commonly (25.5% vs. 20%; p-value 0.4) in patients older than five years of age, and depressed fractional shortening in 11 (13.8%) of the patients.

Laboratory evidence of hyperinflammation at admission was demonstrated in almost all of the patients (Table 4); 98.7% of the patients had C-reactive protein (CRP) levels greater than 5 mg/dL and ferritin levels greater than 400 ng/ml; and 92.5% had an erythrocyte sedimentation rate (ESR) greater than 20 mm/hr. Hypoalbuminemia (albumin level below 35 g/L) was found in 85% of the patients; serum sodium levels below 135 mmol/L were found in 82.5%; and thrombocytopenia (platelets less than 150 x 109/L) was reported in 37.5% of the patients. The significance of the laboratory parameters between the age groups was analyzed using an independent t-test. Only the absolute lymphocyte count (ALC) parameter was found to be statistically significant in patients younger than five years old compared to those older than five years, with a p-value of 0.001. The mean absolute lymphocyte count (ALC) values for the two groups were 1.92 ± 1.18 and 1.10 ± 0.88 respectively.

**Table 4:** Laboratory data in different age groups WBC: white blood cells; ANC: absolute neutrophil count; ALC: absolute lymphocyte count; PCV: packed cell volume; Plt: platelet count; CRP: C-reactive protein; ESR: erythrocyte sedimentation rate; ALT: alanine transaminase; AST: aspartate aminotransferase; NA: sodium; INR: international normalized ratio; mg/dL: milligrams per deciliter; U/L: units per liter

Laboratory data in different age groups										
Parameters		Overall (n=80)		<5 years (n=25)			>5 years (n=55)		p-value	
WBC X10^3 (Mean±SD)		12.29±7.05		13.69±5.57			11.66±7.59		0.235	
ANC X 10^3 (Mean±SD)		10.16±6.52		10.82±5.02			9.86±7.14		0.545	
ALC X10^3 (Mean±SD)		1.36±1.05		1.92±1.18			1.10±0.88		0.001	
PCV % (Mean±SD)		31.28±4.99		30.29±2.74			31.73±5.69		0.234	
PltX10^ (Mean±SD)		202.70±153.27		188.12±155.51			209.33±153.22		0.570	
CRP mg/dL (Mean±SD)		57.06±73.51		61.67±85.01			54.96±68.40		0.708	
ESR mm/hr (Mean±SD)		66.89±29.80		63.24±28.03			68.89±29.80		0.464	
D-dimer (Mean±SD)		4.16±3.50		4.04±3.54			4.22±3.50		0.837	
Ferritin ng/dL (Mean±SD)		960.11±1192.49		727.84±698.27			1065.69±1351.79		0.243	
Fibrinogen (Mean±SD)		4.15±1.41		4.22±1.76			4.12±1.24		0.779	
ALT U/L (Mean±SD)		54.43±69.67		61.80±61.36			51.07±73.42		0.527	
AST U/L (Mean±SD)		74.36±160.69		80.08±117.91			71.76±177.69		0.832	
Albumin (Mean±SD)		3.01±0.49		2.99±0.49			3.03±0.49		0.735	
Na (Mean±SD)		131.19±4.23		131.32±5.18			131.13±3.78		0.852	
INR (Mean±SD)		1.29±0.60		1.43±0.95			1.22±0.34		0.158	

Pharmacological treatments in different age groups are listed in Table 5. More than half (53.7%) of the patients received intravenous immunoglobulin (IVIG) (2 gm/kg) and 73.7% used an anti-inflammatory dose of corticosteroids (methylprednisolone 2 mg/kg/day); six patients with severe and refractory manifestations received methylprednisolone pulse (10-30 mg/kg/day for three consecutive days); and 43.7% of the patients received corticosteroids alone (2 mg/kg/day). Comparing treatment modalities in different age groups showed that using corticosteroids (2 mg/kg/day) was statistically significant in patients older than five years of age (p-value 0.017). During the intensive care unit stay, low-dose aspirin (3-5 mg/kg/day) was administered to 47.5% of patients, and prophylactic low molecular weight heparin was administered to 6.25% of patients. Biological treatment was used with four patients (4.9%) with a refractory severe disease course: tocilizumab was used with two patients (3.7%) and an IL-1 receptor antagonist (anakinra) was used with one patient (1.2%). Inotropic support for hypotension was used with 15 patients (18.7%).

**Table 5:** Pharmacological treatment used in different age groups IVMP: intravenous methylprednisolone; IVIG: intravenous immunoglobulin; LMWH: low molecular weight heparin

Pharmacological treatment used in different age groups				
Steroids	Overall 80 (100%)	< 5 years 25 (31.25%)	> 5 years 55 (68.75%)	p-value
IVMP	65 (81.2)	17 (68)	48 (87.2)	
2mg/kg	59 (73.7)	14 (56)	45 (81.8)	0.017
10mg/kg	3 (4.6)	1 (4)	2 (3.6)	0.681
30mg/kg	3 (4.6)	2 (8)	1 (1.8)	0.229
IVIG	43 (53.7)	16 (64)	27 (49)	0.452
Aspirin	38 (47.5)	13 (52)	25 (45.4)	0.381
LMWH	5 (6.25)	3 (12)	2 (3.6)	0.332
Inotropes	15 (18.7)	5 (20)	10 (18.1)	0.242
Tocilizumab	3 (3.7)	1 (4)	2 (3.6)	0.375
Anakinra	1 (1.2)	0 (0)	1 (1.8)	0.374

## Discussion

A small group of eight patients in England was the subject of the first report on MIS-C [3]. Variable clinical manifestations have been reported since the introduction of MIS-C [6]. In this study, we evaluated 80 patients who met the CDC definition criteria for MIS-C and described the clinical manifestations and the treatment modalities used in the Pediatric Rheumatology Division at Queen Rania Children’s Hospital, Amman, Jordan.

The reported mean age in this study was 84.4 ± 42.2 months, which is similar to that published by Grama et al. [7] and younger than that reported by Sözeri et al., which included patients older than 18 years that were included in our cohort [8].

This study revealed a male predominance (53 male patients, 66.2%, with a male-to-female ratio of 1.9:1); a similar finding was reported by Sözeri et al. [8], while Feldstein et al. reported a lower ratio (1.3:1) [9].

In our study, the nasal swab for SARS-CoV-2 by PCR was positive in 26.2% of the cases, and in 21.1% of the cases, a positive serological antibody assay was reported. The majority of patients in this study (80%) reported positive contact with confirmed COVID-19 cases. Similarly, published studies reported a low incidence of SARS-CoV-2 PCR positivity [10,11,12].

In our study, the most prevalent clinical manifestation was fever, reported in all patients; fever is a mandatory criterion in the CDC and WHO definition criteria for MIS-C. Gastrointestinal manifestations, especially in the abdominal region, are common in MIS-C cases and can be severe enough to require surgery [13]. In our study, abdominal pain was reported in 76.2% of patients, which is similar to that reported by Shabab et al. [14]. But unlike other reports where abdominal pain was reported less frequently [9,13], among other gastrointestinal manifestations in our study were vomiting (61.2%) and diarrhea (42.5%), whereas they were reported in 53.7% and 63% of cases, respectively, by Alharbi et al. [15]. Skin rash (maculopapular) was the second most common clinical presentation and was found in 75% of patients; Toubiana et al. [16] found skin rash in 76% of patients, and Selçuk et al. [17] in 81% of patients.

Neurological manifestations were present in 50% of our patients. These were mild manifestations, including headache, drowsiness, and altered mental status. The most common manifestation was headache (38.7%), with statistical significance found in patients older than five years (p-value < 0.05), which can be attributed to better reporting of headaches in the older age group. Favorable outcomes and complete recovery from neurological manifestations were reported when appropriate treatment was introduced early [18].

Cardiac involvement is common in patients with MIS-C in terms of myocardial dysfunction, arrhythmia, pericardial effusion, coronary artery involvement, and cardiogenic shock [8,9]. In our study, the most common cardiac involvement was pericardial effusion (23.8%), which can be explained by the presence of a diffuse hyperinflammatory process [3]. Evidence of myocardial dysfunction with decreased fractional shortening (FS) was found in 13.8% of patients, while coronary artery dilation was reported in 6.3%. In our study, the presence of pericardial effusion was similar to that reported (24.9%) by Feldstein et al. [9] but higher than that reported (2%, 8.3%) by Mamishi et al. and Valverde et al. [19, 20], respectively. The low incidence of myocardial dysfunction in our study may be explained by the early introduction of corticosteroid treatment [7].

The majority of our patients showed elevated inflammatory markers, including CRP (greater than 5 mg/dL in 98.7%), ESR (greater than 20 mm/hr in 92.5%), and ferritin levels (greater than 400 μg/L in 70%), consistent with immune dysregulation-induced cytokine storm and consistent with those found in the literature [3,21]. Lymphopenia in our study was reported in 72.5% of patients, higher than reported by Mamishi et al. (54%) and (64%) by Lee et al. [19,22]. Lymphopenia was statistically evident in patients younger than five years old (p-value = 0.001), contrary to that reported by Feldstein et al. [9], where lymphopenia was more pronounced in older patients. A previous report by Alkan et al. [23] showed lymphopenia as a marker of MIS-C severity.

Our study reported elevated D-dimer and fibrinogen levels in 98.7% and 58.7% of patients, respectively, which may lead to hypercoagulable states such as deep vein thrombosis and pulmonary emboli. However, none of these complications were reported in our cohort.

Intravenous immunoglobulin (IVIG) and/or corticosteroids are considered first-line treatments according to the first version of the MIS-C treatment recommendations of the American College of Rheumatology (ACR) [24]. In our study, the most common treatment used was corticosteroids (81.2%) (2 mg/kg in 73.7%, 10-30 mg/kg in 9.2%). As stated in a previously published report by Repighan et al., steroids play an important role in controlling cytokine-induced hyperinflammation [3]. IVIG was used in 53.7% of cases. Corticosteroids were used either alone (43.7%) or in combination with IVIG (51.2%). In previous studies by Grama et al. [7], Sözeri et al. [8], and Shabab et al. [14], IVIG was used with 67.6%, 85%, and 96.2% of patients, respectively. The less frequent use of IVIG in our study could be explained by the fact that 67.5% of our patients had mild to moderate disease severity without significant cardiac involvement and showed an initial response to corticosteroids alone, in addition to the shortage of supply and high cost.

Antiplatelet agent prophylaxis is recommended due to platelet activation, thrombocytosis, and endothelial injury [23]. In our study, aspirin was used in 47.5% of cases, which is close to the 50.4% usage figure reported by Ciftdogan et al. [25].

Limitations

One of the limitations of our study is its retrospective nature; furthermore, it is being conducted in a single center rather than being nationally based, which will give more details about MIS-C in Jordan. Another limitation of the study is that we did not include patients older than 16 years of age due to governmental policy on the pediatric age cut-off, which is contrary to WHO/CDC definition criteria.

## Conclusions

MIS-C can present with a wide clinical spectrum and should be considered in the differential diagnosis of patients with prolonged fever, elevated inflammatory markers, and prominent gastrointestinal manifestations. Early diagnosis and prompt initiation of treatment are essential to improving outcomes. In our cohort, we found that abdominal pain and headache are more prominent in patients older than five years, while lymphopenia is more frequent in patients younger than five years. To the best of our knowledge, this is the first report of MIS-C in Jordan, knowing that our hospital is the only tertiary hospital in the country with a pediatric rheumatology division.
